# GraphSNP: an interactive distance viewer for investigating outbreaks and transmission networks using a graph approach

**DOI:** 10.1186/s12859-023-05332-x

**Published:** 2023-05-19

**Authors:** Budi Permana, Scott A. Beatson, Brian M. Forde

**Affiliations:** 1 School of Chemistry and Molecular Biosciences, University of Queensland, Brisbane, QLD Australia; 2 Australian Centre for Ecogenomics, University of Queensland, Brisbane, QLD Australia; 3 University of Queensland Centre for Clinical Research, Royal Brisbane and Women’s Hospital, Brisbane, QLD Australia; 4 Australian Infectious Disease Research Centre, Faculty of Science, The University of Queensland, Brisbane, Australia; 5 Herston Infectious Diseases Institute, Metro North Health, Brisbane, Australia

**Keywords:** SNP distance, Genomic epidemiology, Visualisation, Cluster and transmission analysis

## Abstract

**Background:**

Cluster and transmission analysis utilising pairwise SNP distance are increasingly used in genomic epidemiological studies. However, current methods are often challenging to install and use, and lack interactive functionalities for easy data exploration.

**Results:**

GraphSNP is an interactive visualisation tool running in a web browser that allows users to rapidly generate pairwise SNP distance networks, investigate SNP distance distributions, identify clusters of related organisms, and reconstruct transmission routes. The functionality of GraphSNP is demonstrated using examples from recent multi-drug resistant bacterial outbreaks in healthcare settings.

**Conclusions:**

GraphSNP is freely available at https://github.com/nalarbp/graphsnp. An online version of GraphSNP, including demonstration datasets, input templates, and quick start guide is available for use at https://graphsnp.fordelab.com.

**Supplementary Information:**

The online version contains supplementary material available at 10.1186/s12859-023-05332-x.

## Background

The number of single nucleotide polymorphisms (SNPs) as the proxy of similarity between genomes—commonly referred to as SNP distance—are increasingly used as a measure of relatedness in outbreak studies [1–3]. SNP distances generated from whole genome sequencing (WGS) provide the highest resolution for genotyping, and in combination with other epidemiological data allow investigators to decode transmission dynamics during outbreaks [4, 5].

Current tools that process and visualise SNP distance for outbreak analysis exist mainly as software libraries for programming language environments or lack the combined feature of clustering and transmission reconstruction. R packages, such as Outbreaker [6], Outbreaker2 [7], and Regentrans [8], support both clustering and transmission mapping but require a high level of computer literacy and expertise in order to achieve readily interactive visualisations. In contrast, desktop and web-based visualisation tools such as Phyloviz [9, 10] and CATHAI [11], feature better interactivity but lack support for evaluating distance distribution and reconstructing transmission networks.

To fill this gap, we developed GraphSNP—A **graph**-based and interactive **SNP** distance visualisation tool for cluster and transmission analysis. GraphSNP is built as a simple, ready-to-use standalone visualisation tool to generate interactive charts, networks and trees to rapidly explore outbreak clusters and transmission using SNP distances and associated metadata. Here, we describe and demonstrate the functionality of GraphSNP using examples from previously published datasets.

## Implementation

### Tool overview

GraphSNP implements graph models and algorithms to process and visualise SNP distances within a standalone single page application (SPA) built using React.js [12], AntDesign [13], and Cytoscope.js [14]. The compiled SPA is served using a web server and is accessed via an internet browser. Users can access GraphSNP online at https://graphsnp.fordelab.com or use it offline through a locally-installed web server.

**Fig. 1:**
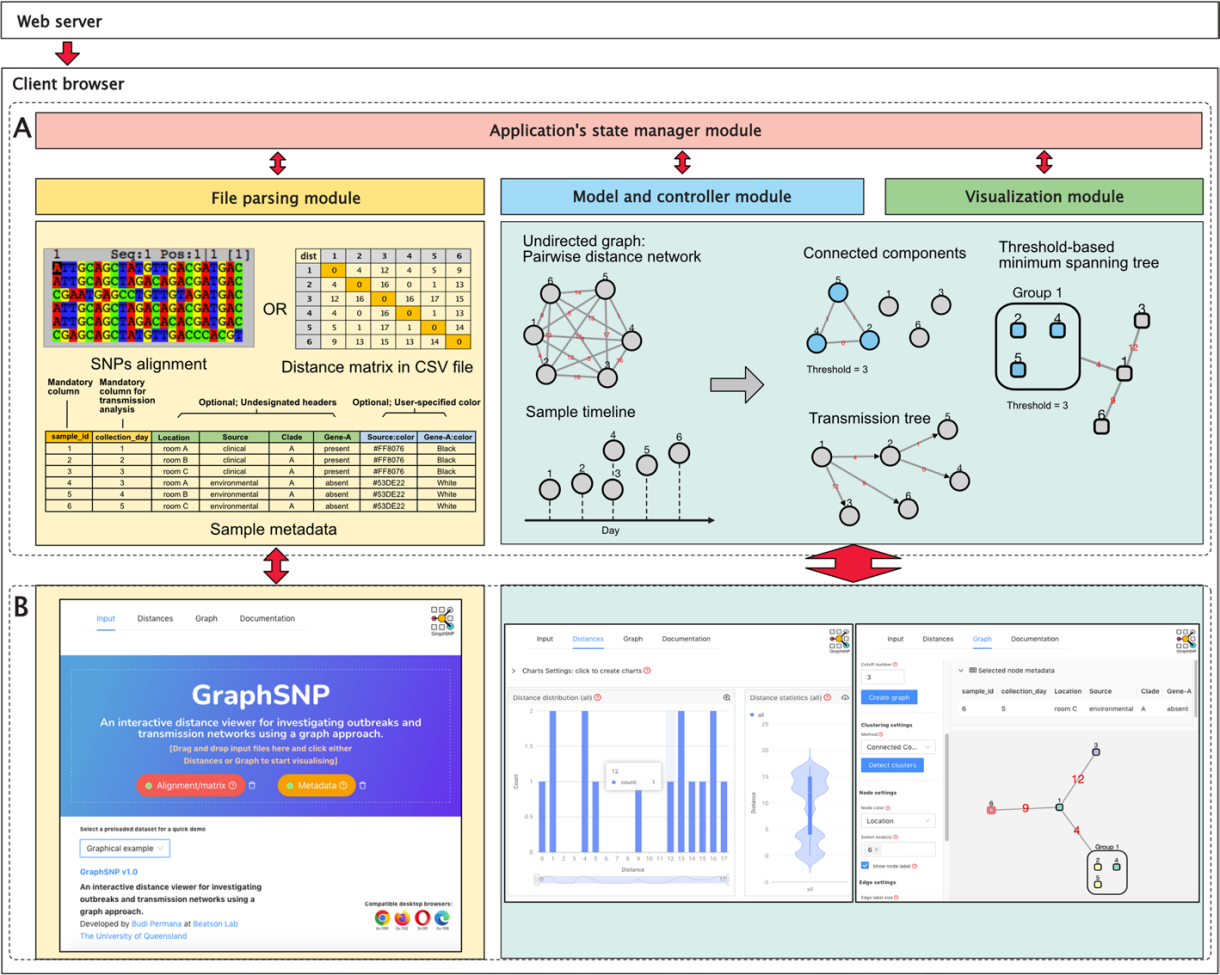
GraphSNP implementation diagram. GraphSNP runs in the client browser to handle the input files, implement graph-based models and algorithms, and visualise the data. **A** Schematic of GraphSNP’s four main processing modules. **B** GraphSNP’s graphical user interface

GraphSNP is comprised of four main modules that handle several interconnected pages: *Input*, *Distances*, *Graph* and *Documentation* (Fig. 1). The *Input* page is a landing page containing a parser module and input placeholder where users upload their data files. The *Distances* and *Graph* pages utilise a graph processing module that allows users to create interactive charts, network, trees and perform cluster and transmission analysis, based on the SNP distance and other related epidemiological data. The *Documentation* page (Additional File 1) provides users with a quick start guide and examples of input files. These pages are all linked together and managed by the application state management module.

GraphSNP generates pairwise Hamming distance from the SNP alignment, visualises it on the network and applies a threshold. Cluster visualisation is performed similar to CATHAI i.e. edges and bridges are removed based on a given threshold to display the connected components. Additionally, GraphSNP provides capability for creating a minimum spanning tree (MST) of the resulted clusters using the Kruskal’s algorithm [15], from kruskal-mst library [16], a transmission tree using the SeqTrack algorithm [6], and membership detection and report using the breadth-first search algorithm [17].

### Using the tool

To start using GraphSNP, users can simply drag and drop the SNP alignment (or distance matrix) and metadata file into the web page. The alignment file must contain a minimum of two fasta-formatted nucleotide sequences of equal length. Alternatively, a symmetric pairwise distance matrix can be uploaded if users precompute the distance using 3rd party tools. The metadata input file is a comma-separated value (CSV) table containing information about the sample with a column “sample_id” referring to the sample identifier that must be identical to the sequence id in the SNP alignment or distance matrix file. An additional column listing sample’s collection time (scaled in days) is required for transmission analysis. Once the input file is loaded, users can create interactive charts, network and trees. Static images and tables can also be downloaded, if required.

### Performance

**Fig. 2:**
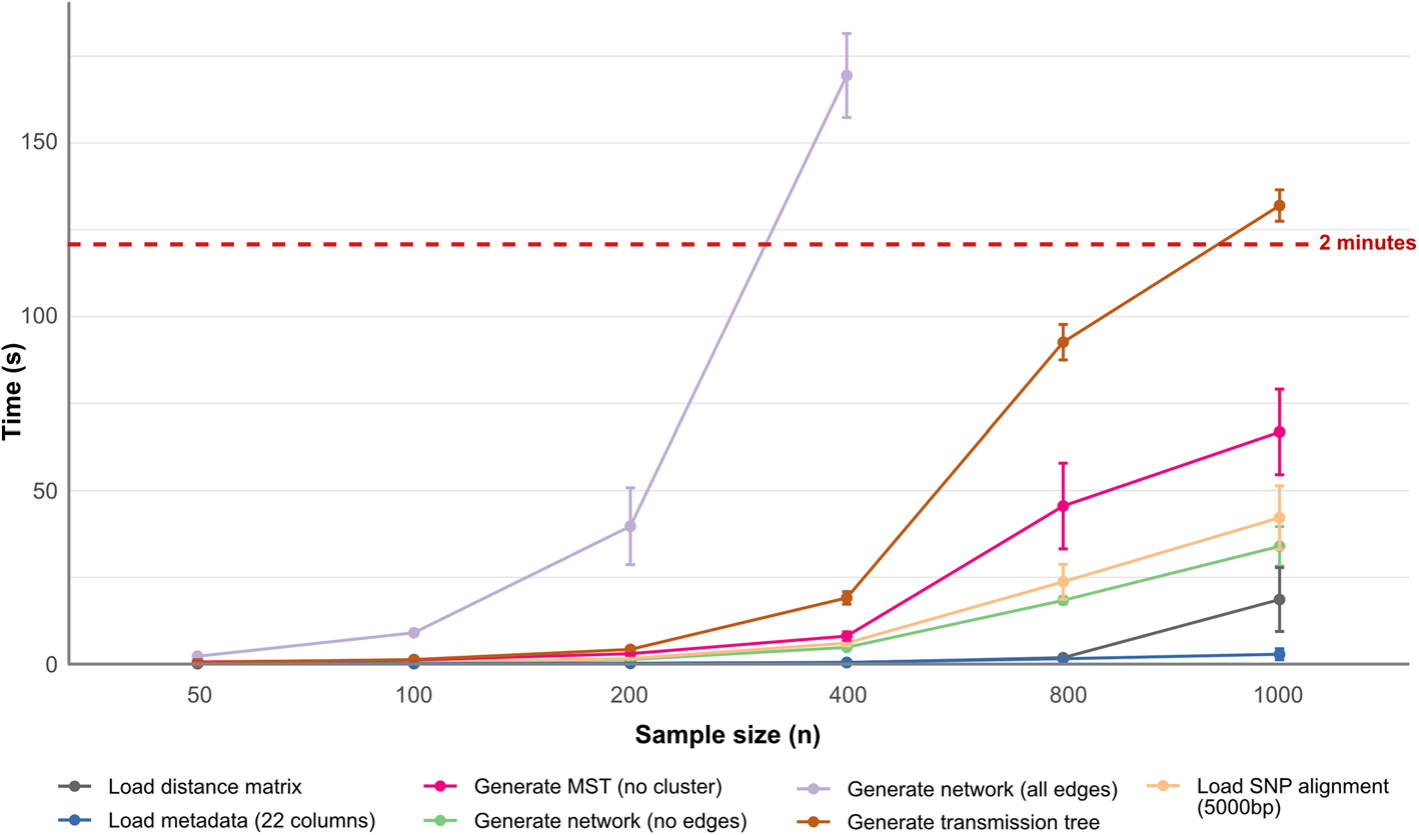
GraphSNP performance on different sample size. The average completion time (seconds; 10 × observations) of GraphSNP’s main functionalities tested using dataset containing 50, 100, 200, 400, 800 and 1000 samples. Tests were performed using Firefox Browser v91.13 on a personal computer with 2.5 GHz CPU, 16 GB RAM, and 1.5 GB VRAM running an OSX system

The average processing time of most key functionalities is < 2 min for datasets containing < 1000 samples (Fig. 2). However, runtime may vary depending on the available computer infrastructure, input, and user defined parameters (e.g. the threshold for identifying clusters and graph layout). Overall, the slowest process is visualising a complete graph of SNP distance (network with all edges being displayed), such as when the threshold is equal to the maximum distance. Generally, GraphSNP performs best on datasets containing less than 500 samples. With higher sample numbers the generated graphs become uninformative, limiting their usefulness to explore outbreaks and transmission events.

### Key features

Compared to the existing tools, GraphSNP's main features focus on the tool's readiness, ease of use, and capacity for both cluster and transmission graph reconstruction. The installation of GraphSNP is minimal (when using it offline) and none (when using it online), providing instant availability for rapid data exploration –similar to the approach adopted by Phandango [18]. Other features, including interactive charts, networks, and trees, are available in GraphSNP. Finally, while SNP distance matrices are used to demonstrate the functionality of GraphSNP, it can display any data that can be presented as a distance matrix (e.g. cgMLST distance matrix [19]). Table 1 summarises the main features of GraphSNP compared to CATHAI [11], Phyloviz 2 [9], and Phyloviz online [10].

**Table 1:** Comparison of GraphSNP’s main features to the existing visualisation tools

GraphSNP main features	GraphSNP v1	CATHAI* [11]	Phyloviz online* [10]	Phyloviz 2* [9]
Doesn’t requires installation	☑		☑	
Support SNP alignment input	☑		☑	☑
Support distance matrix input	☑	☑		
Support metadata input	☑	☑	☑	☑
Visualise pairwise distance graph	☑	☑	☑	☑
Apply threshold to split graph and visualise connected components	☑	☑	☑	☑
Detect cluster and report the membership	☑			☑
Visualise interactive charts for SNP distance distribution and statistics	☑			
Construct and visualise interactive threshold-based cluster MST	☑			
Construct and visualise interactive transmission tree	☑			

*Access of 27–August–2022

### Demonstration dataset

For demonstration purposes, example datasets were created using data from three previously published studies [1, 20, 21]. For the *K. michiganensis* dataset sample metadata was obtained from the manuscript and sequence data for each genome was downloaded from the Sequence Read Archive (SRA). A SNP alignment was created from the sequence read data using the methods described in the study [20]. The *E. faecium* and *K. pneumoniae* SNP distance matrix and metadata were created from SNP distance data and sample information provided in each study's Additional file 1 [1, 21].

## Results and examples

To demonstrate the utility of GraphSNP, we tested it on three previously published datasets [1, 20, 21]. Test datasets are available on the GraphSNP website*.*

### Visualising SNP distance distribution

**Fig. 3:**
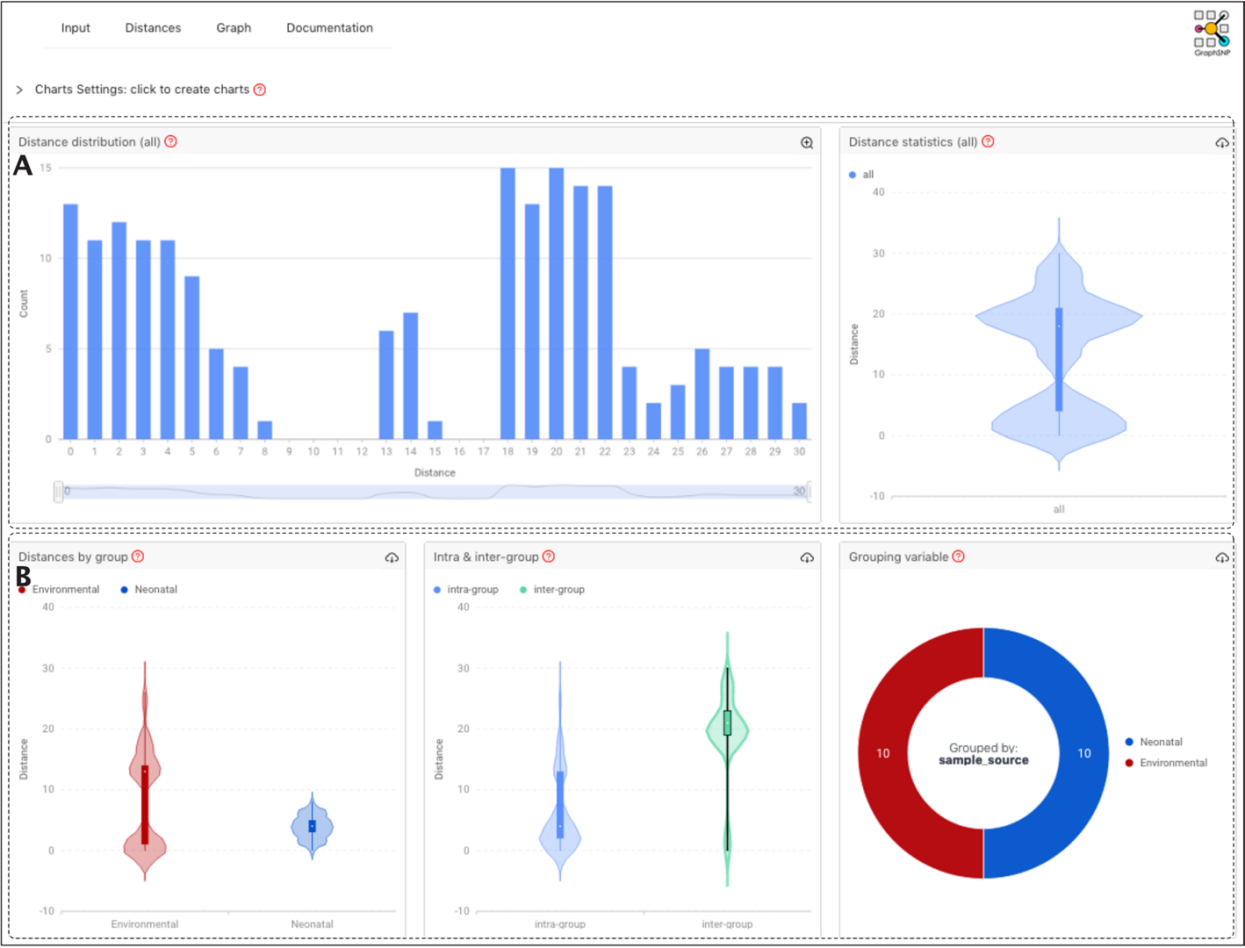
Interactive charts to investigate SNP distance distribution. Interactive charts showing the distribution of all pairwise SNP distances of 20 ESBL-producing *K. michiganensis* [20] (**A**) and SNP distances group based on the sample source (**B**). The distribution of SNP distances within the same group (intra-group) and different groups (inter-group) is also displayed

The distribution of SNP distance can illustrate the relationship between samples and sample groups. GraphSNP provides functionality to visualise these distributions rapidly and interactively. Users can select SNP distances from all samples or sample groups using a dropdown button and visualise its distribution on interactive charts (Fig. 3). The statistics of the distances, including minimum, maximum, mean, median, first and third quartile, are also displayed.

Using an example dataset from Chapman et al. [20], we plotted 190 pairwise SNP distances from 20 ESBL-producing *Klebsiella michiganensis* genomes to display a minimal genetic diversity between the samples (median distance of 18 SNPs, IQR: 4–21 SNPs; Fig. 3A), indicating a probable outbreak, as reported in the study. The distances were distributed into two groups, mainly reflecting the distinction between sample sources: clinical and environmental. The clinical samples have lower SNP distances (median: 4 SNPs, IQR: 3–5 SNPs) than environmental samples (median: 13 SNPs; IQR: 1–14 SNPs), indicating more recent and tighter transmissions in the former than the latter group or contact with the same contaminating environmental reservoir (Fig. 3B).

### Applying SNP distance threshold to identify putative outbreak clusters

Applying the SNP distance threshold is a common approach to rule out unrelated samples from the outbreak when using WGS data. GraphSNP facilitates users to perform this approach visually and interactively. The tool will apply a user-inputted threshold into the SNP distance network and removes 'unrelated' links, leaving only interconnected samples representing a cluster. Generated clusters are highly dependent on the choice of SNP threshold, which can vary between pathogens [3, 22–24]. GraphSNP allows users to explore different thresholds, observe the impact on cluster structure and correlate these relationships with available epidemiological metadata. Furthermore, a range of options are available that allow users to change the network layout, size, colour, and visibility of nodes.

**Fig. 4:**
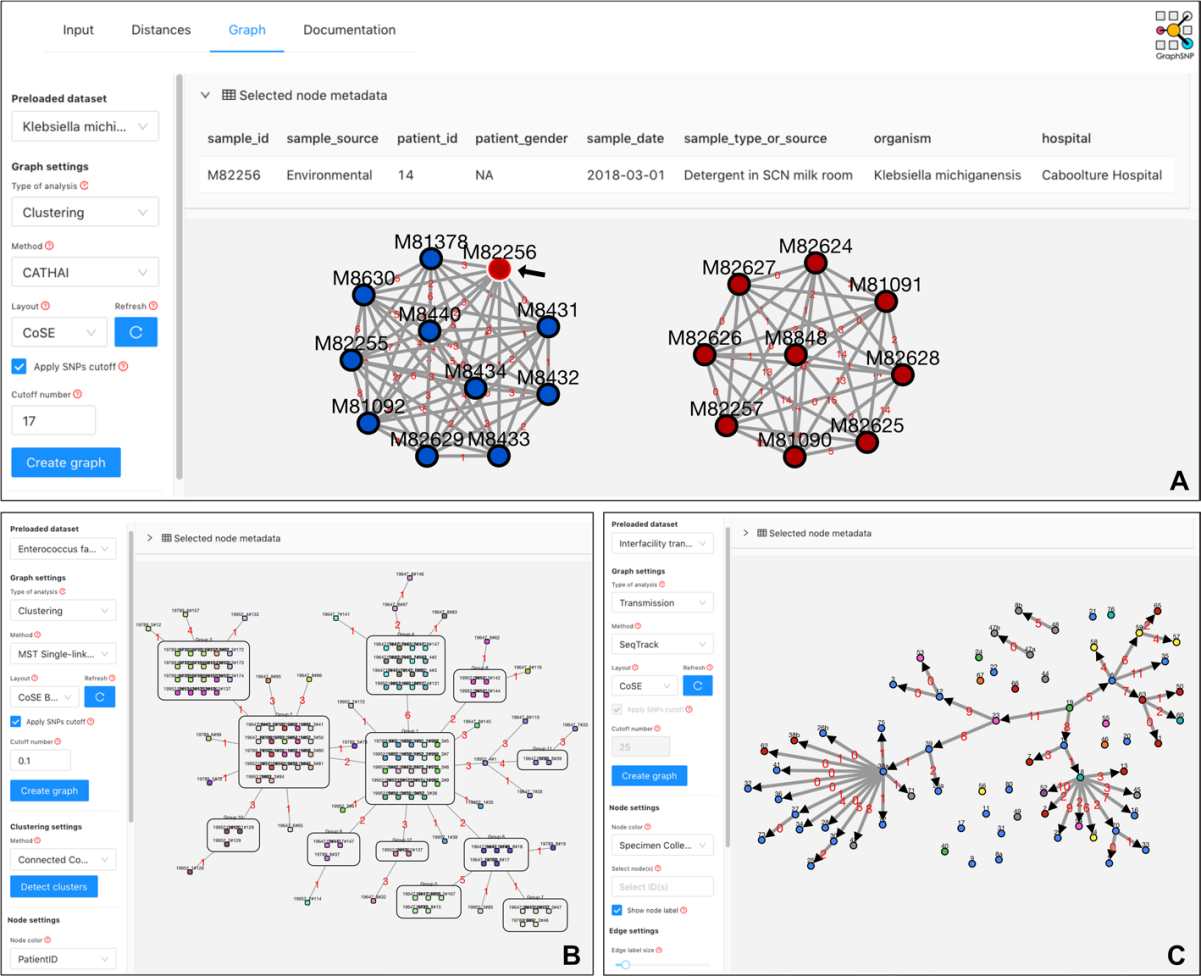
Visualisation of cluster networks, threshold-based minimum spanning tree (MST) and transmission tree. **A** Two split networks represent two clusters of ESBL-producing *K. michiganensis* [20] when applying a threshold of 17 SNPs. Node colour indicates sample source. A black arrow indicates an environmental isolate causing the outbreak. **B** Threshold-based MST displays SNP distances between groups of identical samples (0 SNPs) of Vancomycin-resistant *E. faecium* ST78 subtype 47A [1]. Node colour indicates patient id. **C** A transmission tree shows inter-facility transmission directions of Carbapenem-resistant *K. pneumoniae* [21] based on sample SNP distance and collection timeline. Transmission links with SNP distance greater than 11 SNPs were hidden. Node colour indicates location

Figure 4A demonstrates the resulting clusters from Chapman et al. [20] when applying the thresholds of 17 SNPs to identify two distinct clusters. Colouring the nodes according to the sample source shows that one isolate (M82256), which originated from the detergent bottle (environment sample), was clustered tightly with all clinical isolates within a maximum of 4 SNPs, indicating they have likely been acquired from the same environmental source.

### Visualising distance between clusters using a threshold-based minimum spanning tree

In addition to the standard network visualisation, GraphSNP can also generate a minimum spanning tree (MST) of the resulting clusters after applying the threshold. The tree visualises clusters as compound nodes with edges representing their minimum distance to other clusters or singletons. Figure 4B shows the MST of clusters of genetically identical (SNP distance = 0) Vancomycin-resistant *Enterococcus faecium* ST78 subtype 47A from Gouliouris et al. [1]. The tree shows clusters and singletons were connected by a maximum of 6 SNPs, indicating recent transmission events. Colouring the node according to patient id shows that isolates from the same patient are mainly clustered together, suggesting minimum within-host genetic diversity within the subtype. Compared to the network, a threshold-based MST presents a broader view of the clusters with a faster rendering process, providing better visualisation performance.

### Visualising transmission chains

GraphSNP utilises the SeqTrack algorithm [6] to create a parsimonious transmission tree using sample SNP distances and the date of sample collection. The algorithm originally available in the adegenet R package [25] and was rewritten in JavaScript for GraphSNP to ensure web browser compatibility. The produced tree from this algorithm, combined with additional edge filtering to mask unlikely links beyond a predefined threshold, can be used to visualise the potential transmission chains among samples. Using an example dataset from Spencer et al. [21], GraphSNP reproduces and visualises inter-facility transmissions of Carbapenem-resistant *Klebsiella pneumoniae* (Fig. 4C). Consistent with the study, the tree shows the transmission directions between samples with low genomic diversity (SNP distance ≤ 11 SNPs) across several healthcare facilities.

### Offline use of GraphSNP with preloaded datasets

While GraphSNP is readily available online, the tool can also be accessed offline using a locally-installed web server. When using offline mode, datasets can be preloaded into GraphSNP and stored for repeated use. Additionally, running GraphSNP locally allows for easier integration with 3^rd^ party tools or pipelines and the seamless flow of necessary input files into GraphSNP. Detailed instructions on how to set up GraphSNP offline with customised preloaded datasets are provided in the *Documentation* page.

### Limitations

As a standalone, client-side application running entirely on the browser, GraphSNP has several limitations. First, performance largely depends on the specifications of the computer used to access GraphSNP. Second, it is not optimised to process and visualise large datasets (for example, visualising a graph containing more than 500 samples and SNP alignments over 5000 bp). In such cases, users may be advised to use tools utilising separate processes, such as CATHAI [11] that use server-side precomputation for its graph and layout. Third, a parsimonious tree generated in GraphSNP does not convey the uncertainty in transmission events, making it unsuitable for complex outbreak scenarios where the directionality of transmission should be depicted through probabilities [6, 26]. Finally, while visualising SNP distance and metadata are relatively fast, it is worth noting that SNP alignments need to be computed outside of GraphSNP using 3^rd^ party tools.

## Conclusions

GraphSNP allows users to visualise SNP distances for investigating outbreak clusters and transmission interactively. The intuitive web application design, minimal installation and configuration steps, make it easy to use for rapid exploratory analysis. GraphSNP is freely available for both online and offline use.

## Availability and requirements

Project name: GraphSNP

Project home page: https://github.com/nalarbp/graphsnp

Operating system(s): Platform independent

Programming language: JavaScript

Other requirements: None for online use, a local web server for offline use

License: MIT

Any restrictions to use by non-academics: None

## Supplementary Information

**Additional file 1** The Documentation page provides users with a quick start guide and examples of input files.

## Data Availability

All data generated and used for demonstration purpose of GraphSNP are included within the tool and are available in the GitHub repository (https://github.com/nalarbp/graphsnp).

## Abbreviations

CATHAI: Cluster analysis tool for hospital-associated infections
CSV: Comma-separated value
MST: Minimum spanning tree
SNP: Single nucleotide polymorphism
SPA: Single page application
WGS: Whole genome sequencing
