# Supplementary material for: GraphSNP: an interactive distance viewer for investigating outbreaks and transmission networks using a graph approach
Source: BMC Bioinformatics. 2023 May 19;24:209. doi: 10.1186/s12859-023-05332-x (PMC10199467; doi:10.1186/s12859-023-05332-x)
Supplement: Supplementary file 1 — Additional file 1 The Documentation page provides users with a quick start guide and examples of input files. [file 12859_2023_5332_MOESM1_ESM.pdf]

GraphSNP

GraphSNP USER MANUAL

Budi Permana

v.2023.02.22

Using GraphSNP

GraphSNP is a single page application (SPA) visualisation tool that runs on the browser. Users can visualise and explore data by loading their input files or setting up multiple projects (available on offline use only) for multiple input datasets.

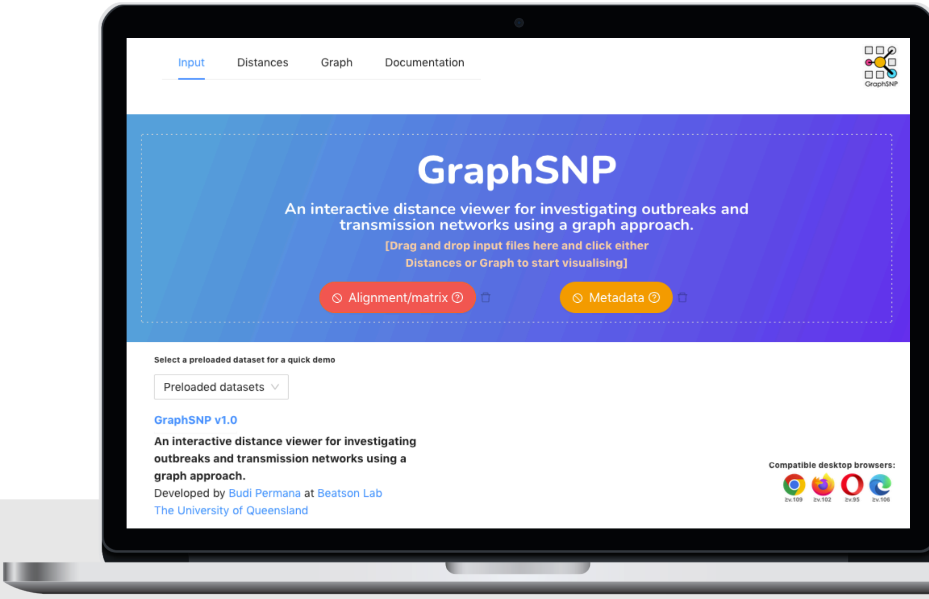

https://**graphsnp.beatsonlab.com/**

Tested and compatible with

≥v.109 ≥v.102 ≥v.95 ≥v.106

! Browser compatibility issue

Some older versions of browsers (e.g. IE, Safari) have trouble displaying the network, resulting in an empty display. The latest version of Google Chrome and Firefox provide the best experience.

Use it online

GraphSNP is deployed in https://graphsnp.beatsonlab.com for online use. Users can visit the web page using modern browsers (e.g., Google Chrome, Firefox, Microsoft Edge), drag and drop the input files, and instantly perform interactive data visualization and analysis.

Use it offline

Users also can use GrapSNP offline by serving it through a local HTTP server. GraphSNP SPA can be downloaded from https://github.com/nalarbp/graphsnp/build/.

Example of serving GraphSNP using HTTP-server “*serve*” tool

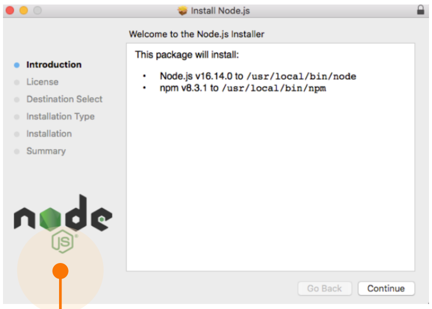

Install Node.js
(available at https://nodejs.org/en/)

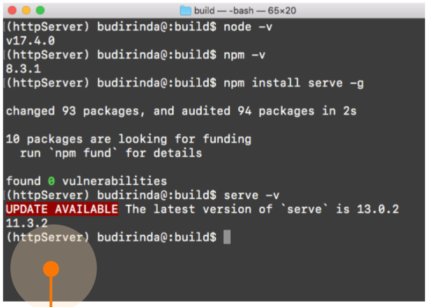

Install serve via npm
(npm install serve -g)

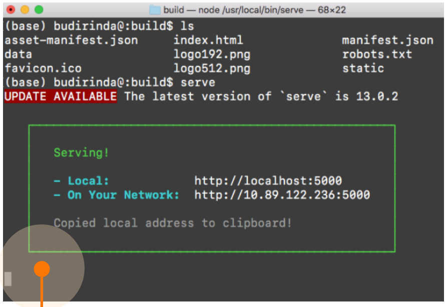

Run the HTTP server
(serve .)

Input Files

SNPs alignment

A text file containing a minimum of two equal lengths of fasta-formatted nucleotide sequences. Gap and non-ATGC characters will be omitted from the pairwise distance calculation (see page 7 for more detailed description).

Example SNPs alignment input *(sample.fasta)*

```
>1
ATTGCAGCTATGTTGACGATGAC
>2
ATTGCAGCTAGACAGACGATGAC
>3
CGAATGAGCCTGTTGTAGATGAC
>4
ATTGCAGCTAGACAGACGATGAC
>5
ATTGCAGCTAGACACACGATGAC
>6
CGAGCAGCTATGTTGACCCACGT
```

Sample ID in fasta header

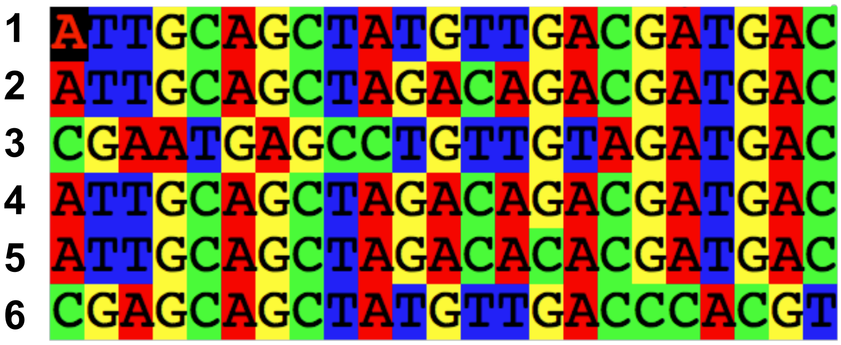

Pairwise distances matrix

Example of pairwise SNP distances matrix *(sample_matrix.csv)*

dist	1	2	3	4	5	6
1	0	4	12	4	5	9
2	4	0	16	0	1	13
3	12	16	0	16	17	15
4	4	0	16	0	1	13
5	5	1	17	1	0	14
6	9	13	15	13	14	0

Matrix in CSV format

```
dist,1,2,3,4,5,6
1,0,4,12,4,5,9
2,4,0,16,0,1,13
3,12,16,0,16,17,15
4,4,0,16,0,1,13
5,5,1,17,1,0,14
6,9,13,15,13,14,0
```

User can also input the pairwise distances matrix instead of SNP alignment. The symmetric matrix should be written in comma-separated value (CSV) format.

Metadata

A table contains information about the isolates or sample, written in CSV format. Critical requirements including: mandatory headers, no duplicated records in column **sample_id**. Column **collection_day** is required for transmission analysis.

Mandatory column (sample_id); Mandatory column for transmission analysis (collection_day); Optional; Undesignated headers (Location, Source, Clade, Gene-A); Optional; User-specified color (Source:color, Gene-A:color)

sample_id	collection_day	Location	Source	Clade	Gene-A	Source:color	Gene-A:color
1	1	room A	clinical	A	present	#FF8076	Black
2	2	room B	clinical	A	present	#FF8076	Black
3	3	room C	clinical	A	present	#FF8076	Black
4	3	room A	environmental	A	absent	#53DE22	White
5	4	room B	environmental	A	absent	#53DE22	White
6	5	room C	environmental	A	absent	#53DE22	White

Main Interface

Page navigation

Navigation menu to let you jump between pages: *Input, Distances, Graph, and Documentation.*

Metadata table

Let you display metadata associated with selected node(s).

<u>page *Graph*</u>

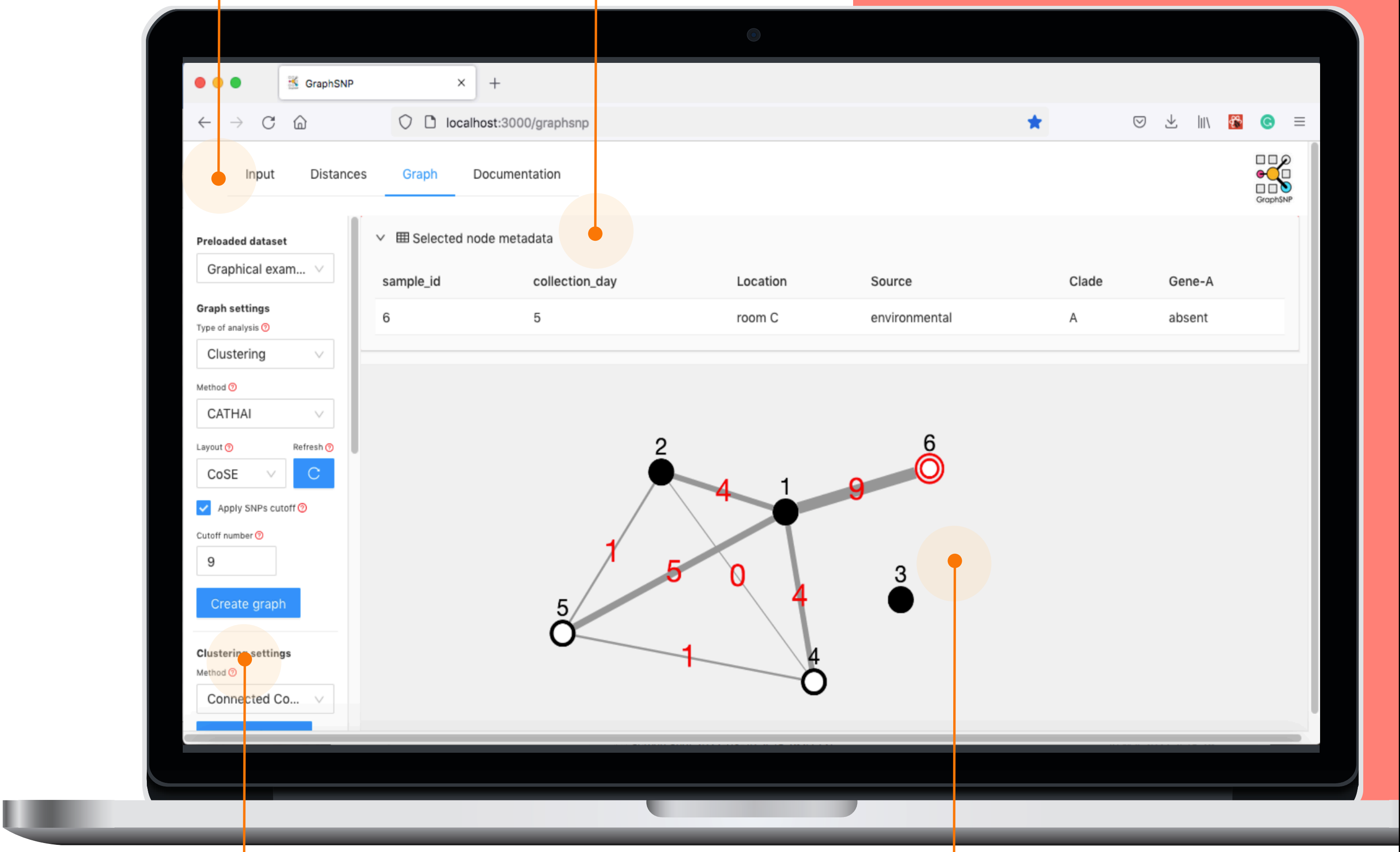

Sidebar settings

A sidebar menu provides you a control to adjust the visualisation.

Graph visualisation window

A window container where the interactive graph is being rendered.

<u>page *Input*</u>

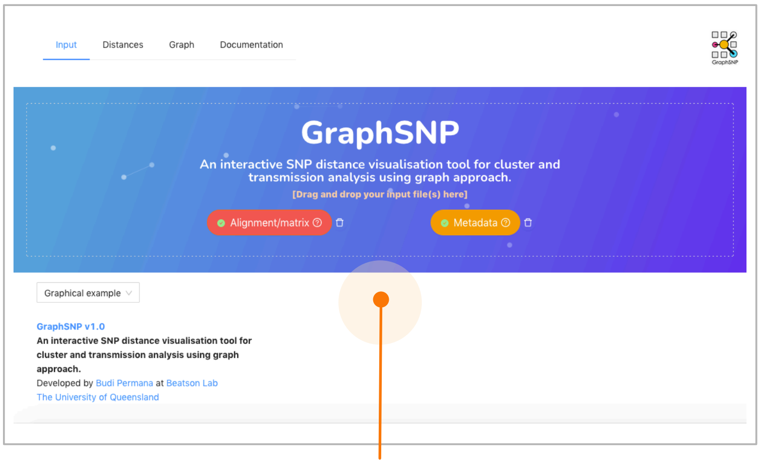

<u>page *Distances*</u>

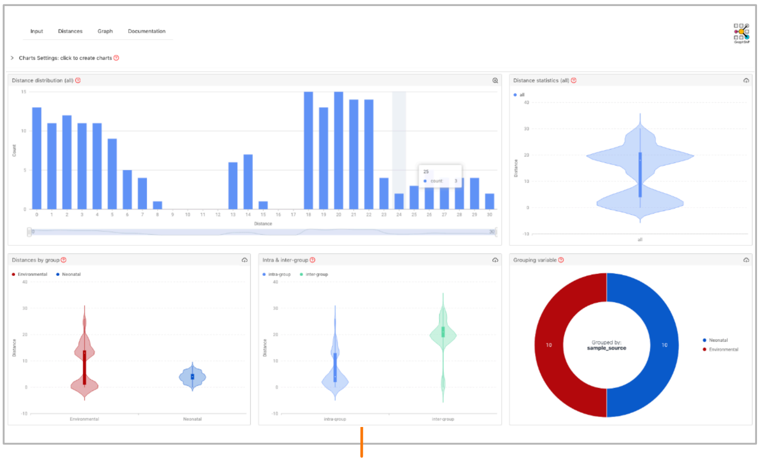

Input placeholder

Drag and drop your input files here.

Chart visualisation window

A container where charts showing pairwise distances count and statistics is being rendered.

Cluster analysis

Cluster analysis and visualization can be performed in five simple steps: loading input files, select clustering as the type of analysis, select the clustering method, construct the graph, and detect/report clusters from the graph.

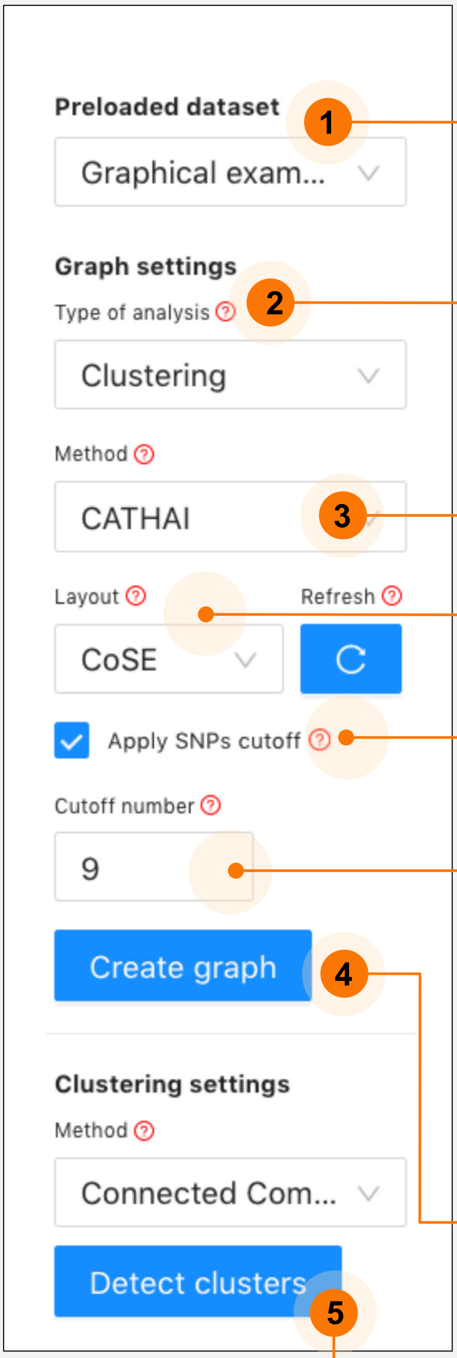

Load input files

Select Clustering

Select reconstruction method

Network: *an undirected Graph based of pairwise distances. Node represents individual isolate. Edge represents distance.*

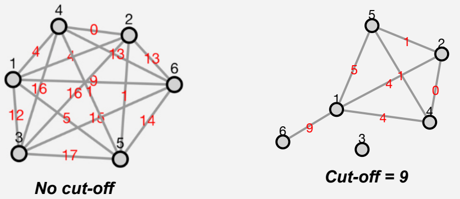

No cut-off

Cut-off = 9

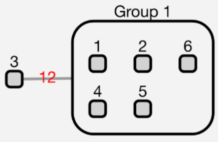

Cut-off = 9

Threshold-based minimum spanning tree (MST): *Produce an MST of the identified cluster/s. Individual node represents isolate, while group node represents cluster given the threshold. Edges represents a minimum distance between cluster/s or between or to singleton/s.*

Select which layout to display the Graph.

Layout options are based on Cytoscape.JS layouts (https://js.cytoscape.org/). *These algorithms (CoSE, fCoSE, CoSE Bilkent, and spread) determine the position of nodes and edges in a graph to provide a better graph visualisation, such as avoiding overlap and maintaining a clear structure of the graph.*

Apply a cutoff number to limit the maximum pairwise distance value to be displayed

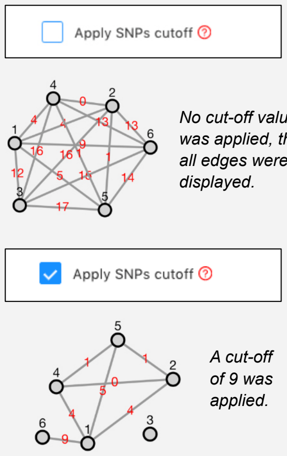

No cut-off value was applied, thus all edges were displayed.

A cut-off of 9 was applied.

Set cut-off value

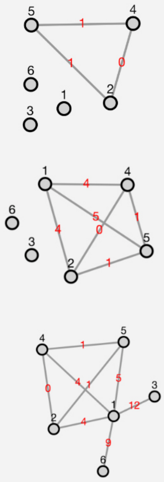

Cut-off = 1

Cut-off = 5

Cut-off = 12

Create Graph

Identify cluster

A cluster is defined by identifying node within the connected component/s, which created by removing edges greater than the threshold.

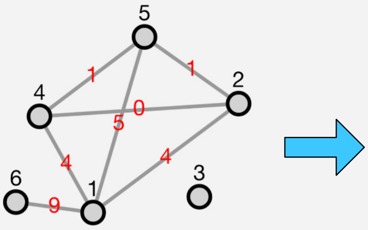

sample	clusterID
1	1
2	1
4	1
5	1
6	1
3	na

*The clustering result can be downloaded as **a CSV file**.*

Transmission analysis

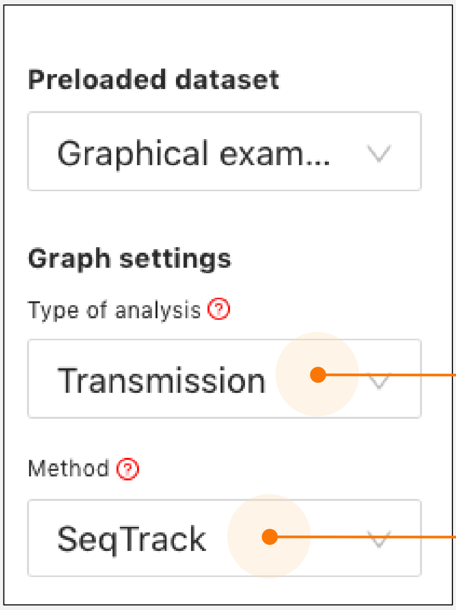

Performing transmission analysis is similar to cluster analysis. Users only need to select Transmission instead of Clustering. Currenltly, only one method is implemented: SeqTrack [1]

1. Jombart, T., et al., *Reconstructing disease outbreaks from genetic data: a graph approach*. Heredity (Edinb), 2011. **106**(2): p. 383-90.

Select Clustering

Select reconstruction method

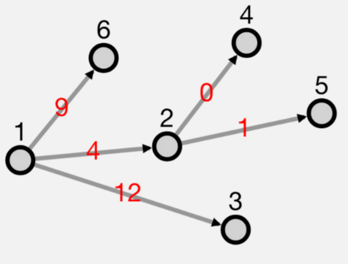

Most parsimonious transmission tree created using SeqTrack algorithm.

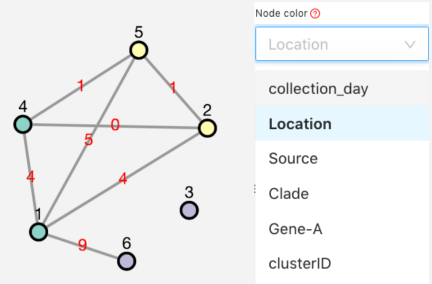

Color nodes by the selected column in metadata or by the clustering result.

Here, we color the nodes based on Location column in metadata.

Show or hide node's label.

Hide label *Show label*

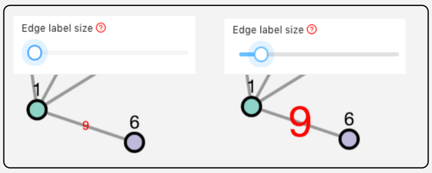

Change edge label size

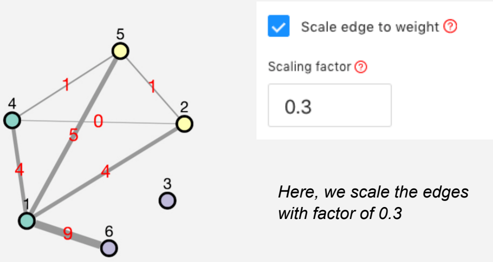

Here, we scale the edges with factor of 0.3

Change the thickness of the edge according to its weight. (e.g., the higher the SNP distance the thicker the line).

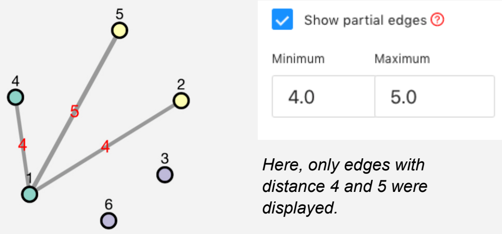

Here, only edges with distance 4 and 5 were displayed.

Only show edges which have weight within the specified range (min to max) (Note: It doesn't remove the edges but only hide it to the background)

Download Graph image (SVG or PNG) or Graph file (DOT) or clustering result (CSV)

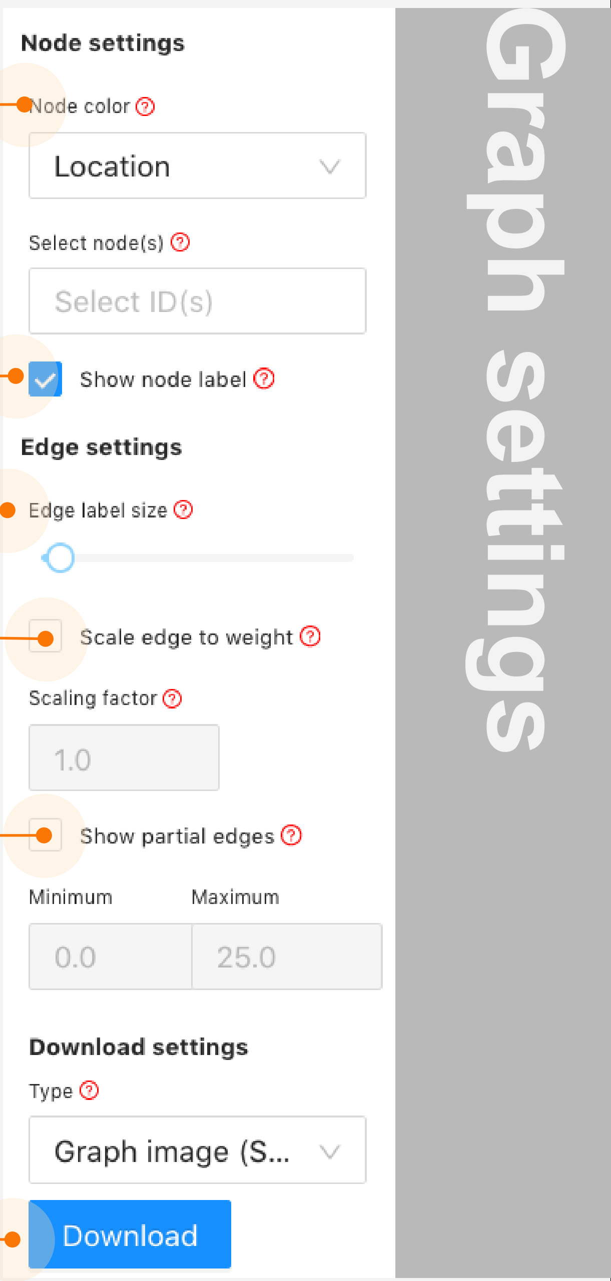

Setting up preloaded dataset

When users use GraphSNP offline, they can set up multiple preloaded datasets. This feature allows users to 'permanently' link their input files to GraphSNP, avoiding the need to re-inputting their input files every time the browser refreshed.

Example of directory tree of GraphSNP preloaded datasets

```
GraphsSPA or build directory
├─ data
│  └─ preloaded
│     ├─ 1
│     │  ├─ snpAlignment.fasta
│     │  └─ metadata.csv
│     └─ 2
│        ├─ distanceMatrix.csv
│        └─ metadata.csv
└─ project.json
```

1 **Go to directory of *data* in GraphSNP SPA**

2 **Create a new directory in *preloaded* directory then add the input files.**

3 **Update project.json file**

Example project.json content

Add the dataset ID and input files path to project.json and save the file.


```
{
  "projects": [
   {
    "id": "1",
    "name": "Dataset 1: Graphical example",
    "matrixOrAlignment": "alignment",
    "snpDistance": "./data/preloaded/1/snpAlignment.fasta",
    "metadata": "./data/preloaded/1/metadata.csv"
   },
   {
    "id": "2",
    "name": "Dataset 2: NCBI Cluster of VREfm ST78",
    "matrixOrAlignment": "matrix",
    "snpDistance": "./data/preloaded/2/distanceMatrix.csv",
    "metadata": "./data/preloaded/2/metadata.csv"
   }
  ],
  "description": "This JSON file describes preloaded datasets to be rendered in the landing page. The path of these files must be written with directory 'public' as the root (e.g. ./data/ means 'data' is inside directory 'public'"
}
```


4 **Datasets is listed in GraphSNP *input* page**

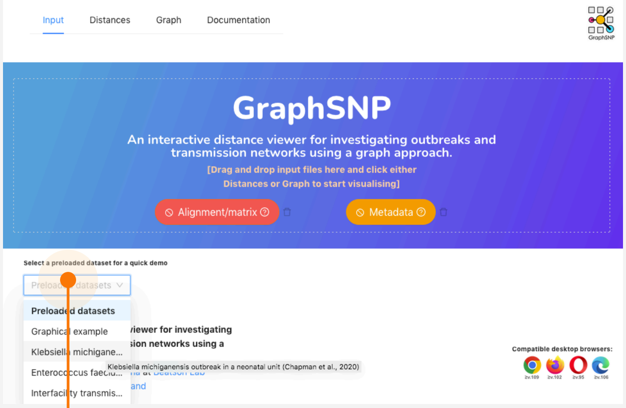

Click the preloaded dataset dropdown button and select dataset of interest and GraphSNP will automatically load the input files.

Extra

1. How does GraphSNP determine the Hamming distance between two sequences?

```
C T G - C N A
| | | | | | |
A G A A T C A
1 + 1 + 1 + 0 + 1 + 0 + 0
```

Given two strings of equal length, GraphSNP counts the number of mismatches (differences) between the corresponding positions containing A, T, G, or C characters. Positions that containing any other characters are omitted from the counting.

Total distance = 4

2. What can I do if the GraphSNP distance calculation is not appropriate for my data?

GraphSNP also support a distance matrix input. It is recommended that users compute the distance using their preferred method and save the result as a distance matrix file. The following are several examples to consider:

#Using ape R pacakge

```
library(ape)
#read alignment file
seq <- read.dna('snps_alignment.fasta', format = "fasta")
#convert to distance matrix
dist <- as.data.frame(dist.dna(seq, model = 'N', as.matrix = T))
#adjust rownames
dist_GraphSNP <- cbind('rowCol' = rownames(dist), dist)
#write out distance matrix to a CSV file
write.csv(dist_GraphSNP, file = "GraphSNP_distanceMat.csv",
row.names = F)
```

#Using snp-dists

```
#Create tool environment and
install snp-dists (https://
github.com/tseemann/snp-dists)
conda create -n snp_dists_env -c
conda-forge snp-dists
#Activate the environment
conda activate snp_dists_env
#Generate distance matrix and
output a CSV file
snp-dists -c
snps_alignment.fasta >
GraphSNP_distanceMat2.csv
```

3. How does GraphSNP generate a threshold-based cluster MST minimum spanning tree?

A threshold-based cluster MST refers to an MST of the identified cluster/s given a threshold, meaning this MST was constructed **AFTER** the cluster/s is defined. It involves 2 main steps: Identification of cluster/s (given a threshold) and constructed the MST between those clusters and or singletons.

A complete graph

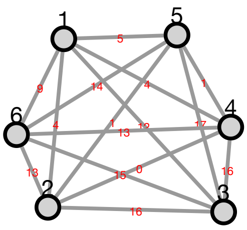

A complete graph built from paiwise distance matrix.

Step 1. Cluster definition (cut-off = 9)

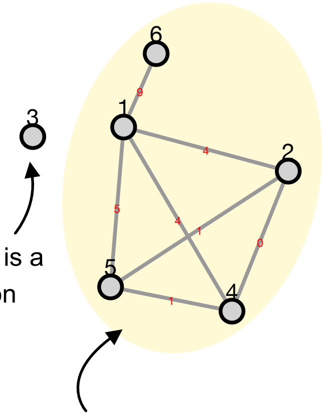

Node 3 is a singleton

Node 1, 2, 4, 5, and 6 are assigned into one cluster (one connected component) using breadth-first search algorithm to identify connected components.

Step 2. MST construction

Kruskal's algorithm is applied to evaluate minimum distance edges between clusters, between singletons and between cluster to singletons to create an MST.

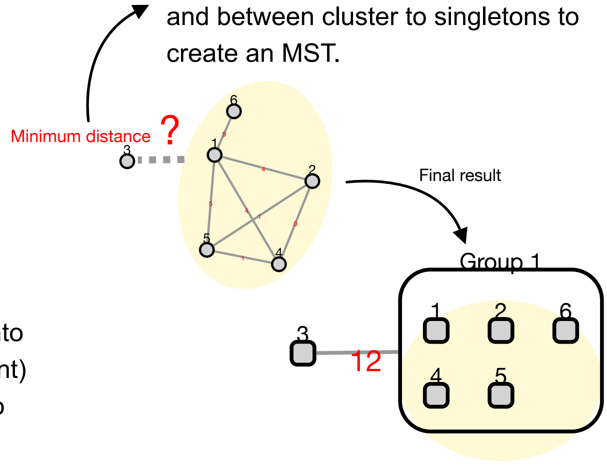

THANK YOU

for reading this manual

Thanks to all awesome web frameworks and libraries run on the background, GraphSNP is now up and running and available worldwide. The following are some of the core libraries used by GraphSNP:

react
d3
antd
cytoscape
cytoscape-svg
redux
react-color
lodash
moment
moment-range
...
